# From Passive Listeners to Active Learners: Changing Student Preferences in Small Group Learning for the MBBS Curriculum

**DOI:** 10.7759/cureus.73289

**Published:** 2024-11-08

**Authors:** Magna Manjareeka

**Affiliations:** 1 Physiology, Kalinga Institute of Medical Science, KIIT (Kalinga Institute of Industrial Technology) Deemed to be University, Bhubaneswar, IND

**Keywords:** assessment, medical education, multiple choice question, self directed learning, student seminar

## Abstract

The shift toward student-centered learning in medical education has become increasingly apparent in recent years, particularly in small group learning (SGL) within the MBBS curriculum. Traditionally, SGL has been teacher-led, but data from three polls conducted across three MBBS batches (2019, 2022, and 2023) highlight a notable shift in student preferences toward more active learning methods. While the 2019 batch overwhelmingly favored teacher-led explanations, the 2022 and 2023 batches showed a growing preference for self-directed learning, with “Self-reading and Doubt Clearing” becoming the most popular mode by 2023. Monthly assessments have also seen fluctuating support, with the 2023 batch reviving interest in regular testing as a tool for success. Notably, the preference for Multiple Choice Questions (MCQs) over traditional long-form questions in regular assessments has risen, reflecting a demand for efficient, objective testing methods that better align with professional exam formats. These trends suggest that medical educators must adapt to a more student-centered, autonomous learning environment while balancing the need for structured assessments. The editorial explores how these changes in student preferences have far-reaching implications for the future of medical education, calling for an approach that emphasizes autonomy, regular feedback, and a practical, outcome-focused curriculum.

## Editorial

In recent years, the approach to medical education has undergone a profound transformation, driven by changes in both curriculum and student preferences. One area that reflects this change most evidently is small group learning, a key component in MBBS programs. Small group learning or SGL has been known to be of great importance for improving understanding of the subject matter and improvement of clinical skills [1]. Small group learning should ideally have an optimal size of six people. However, it is not defined by the size of the group but must include the active participation of everyone involved [2]. Traditionally, small group learning relied heavily on teacher-centered methods where instructors would lead discussions, explain key topics, and clarify doubts. However, data collected from a WhatsApp poll from three MBBS batches (2019, 2022, and 2023) suggest that students are increasingly favoring a more active role in their learning process.

This editorial delves into the evolving student perspective on small group learning, based on the findings from three polls conducted across these batches. The results reveal a fascinating shift in learning preferences, assessment choices, and the types of evaluation students desire. These changes have far-reaching implications for the future of medical education and offer valuable insights for educators looking to refine their teaching methods.

Poll 1: preferred mode of small group teaching-from teacher-led to student-led

The first poll asked students, "Which mode of teaching would you prefer in Small Group Teaching (Tutorials)?", with four options and the choice of selecting multiple options: Teacher explaining the topic, On-the-spot assignments, Student presentations, and Self-reading with doubt-clearing. The number of students per small group was 38 of the 150 students in the 2019 batch and 63 of the 250 students in the 2022 and 2023 batches. The polls were collected in 2019, 2022, and 2023 during the first professional year of the respective batches.

The 2019 batch, a cohort accustomed to more traditional teaching methods, overwhelmingly preferred the teacher-centered approach, with 72.8% out of 162 responses choosing "Teacher explaining the topic". This demonstrates a strong inclination toward a structured learning environment where the teacher is the primary source of knowledge. This has been, however, shown to be a less effective model of learning in several studies [3]. Of the 162 responses, 19.75% believed that "Self-reading with doubt clearing" was the most suitable model.

However, the trend shifts dramatically by the time we reach the 2023 batch. In the 2022 batch, 66.6% of the 48 responses preferred teacher-led explanations, with a noticeable rise in interest in alternative methods like presentations and self-reading. By 2023, the most popular option became "Self-reading and Doubt Clearing" with 72.22% of the 108 responses choosing this method, while teacher-led explanations saw a sharp decline, garnering just 23.14% of the 108 responses. This shift underscores a growing desire for autonomy in the learning process, with students favoring self-directed learning over passive listening.

Interestingly, the appeal of on-spot assignments and student presentations remained low across all batches, indicating that while students want more control over their learning, they do not necessarily want the added pressure of constant evaluations or peer presentations in small group settings. In 2019, 2022, and 2023 batches, 0%, 10.4%, and 0.9% out of 162, 48, and 108 responses respectively voted for on spot assignments. While for student presentation, the votes were 7.4% of 162 responses from 2019, 6.25% of 48 responses from the 2022 batch, and 3.7% of 108 responses from the 2023 batch. The data shows that students are moving toward self-paced learning but still value the instructor's role as a facilitator for clarifying doubts.

Poll 2: monthly assessments as a tool for success

The second poll asked students, "Can monthly assessments help you score better in your professional exams?", with two options "yes" and "no" and only a single option allowed. This question highlights the role of frequent group-wise evaluations in reinforcing learning, a practice that has gained traction in medical education in recent years.

Among the 2019 batch, 96.99% out of 133 responses voted "Yes" and the rest of the responses voted "No", suggesting that regular assessments were seen as a helpful tool for revising content and preparing for final exams. This sentiment, however, experienced a drop with the 2022 batch, where only 66.1% of the 59 responses believed monthly assessments would improve their performance. Thirty-three point eight-nine percent (33.89%) of the 59 responses chose "no" as their option. This reduction could reflect growing concerns about assessment fatigue and anxiety, with students becoming wary of over-assessment, a common issue in rigorous programs like MBBS [4,5].

Nevertheless, the 2023 batch saw a resurgence in support for monthly assessments, with 88.42% of the 95 responses voting "Yes" and 11.57% of 95 responses voting "No". This fluctuation might indicate that while there is some resistance to frequent evaluations, many students still appreciate the value of regular testing, provided the assessments are well-designed and aligned with their study goals.

Poll 3: the ideal format for monthly assessments

The third poll asked students, "If Yes, then which type of questions will you prefer in your monthly assessment exams?", with 3 options namely "Proper questions as in professional exams", "MCQ (Multiple Choice Questions)" and "Short Answer Questions" with the provision of a selection of multiple options. This aimed to explore the types of questions that students feel best help them prepare for their professional exams.

In the 2019 batch, the preference was overwhelmingly for "Proper questions as in professional exams" with 71.43% of the 154 responses selecting this option. Of the 154 responses, 27.27% voted for "MCQ or Multiple Choice Questions". This reflects a strong alignment between regular assessments and final exam formats, as students believed that being tested in the same way would better prepare them for the real thing. A similar trend was observed in the 2022 batch. In 2022, 69.56% and 30.43% out of the 46 responses voted for "Proper questions as in professional exams" and "MCQ", respectively. 

However, this trend shifted with the 2023 batch. By 2023, a majority (71.42% of the 98 responses) opted for Multiple Choice Questions (MCQs), which are often seen as a more efficient way of testing knowledge without the burden of long-form writing. Out of 98 responses, 27.55% voted for "Proper questions as in professional exams" in the 2023 batch. The preference for MCQs could stem from the increasing focus on objective testing methods in medical exams like the National Eligibility cum Entrance Test (Postgraduate) (NEET PG), Institute of National Importance Combined Entrance Test (INI-CET), and the proposed National Exit Test (NExT Exam), where accuracy and time management are critical. The votes for "Short Answer Questions" remained low across all the batches with 1.29% out of 154 responses, 2.1% out of 46 responses, and 1% out of 98 responses for the 2019, 2022, and 2023 batches, respectively.

The dramatic decline in support for "Proper questions as in professional exams" among the 2023 batch (27.55% out of 98 responses) suggests that students are prioritizing efficiency and practicality over depth in regular assessments. They seem to prefer a system that allows for quick knowledge checks through MCQs while reserving the more detailed, written responses for final exams.

The changing landscape of small group learning and assessments

The results of these three polls reveal several important trends that could shape the future of small group learning in MBBS curricula:

Shift Toward Self-Directed Learning

As seen in the first poll, students are increasingly moving away from teacher-led tutorials and are seeking more autonomy through self-reading and doubt-clearing sessions. This shift may reflect a broader trend in education where learners want more control over their educational journey, utilizing digital resources and self-study techniques. However, the need for clarification from instructors remains essential, highlighting the ongoing importance of skilled facilitation in small-group settings.

Balanced Approach to Assessments

The mixed response to monthly assessments indicates that students recognize the value of frequent testing, but there is also a clear demand for balance. The rise in support for monthly assessments in the 2023 batch suggests that students appreciate the role of regular testing, but the format and frequency must be carefully managed.

Preference for MCQs in Regular Testing

The growing preference for MCQs in monthly assessments signals a shift toward more streamlined and efficient testing methods. Students likely appreciate the objective nature of MCQs, which allow for quicker feedback and a more manageable workload compared to long-form questions. Another probable cause of students preferring MCQ pattern questions could be that all the national qualifying exams like NEET-PG etc follow this pattern. This shift has significant implications for how educators design assessments, as they must ensure that MCQs are rigorous enough to test deep understanding while also offering the practical benefits students seek.

The editorial, however, has a limitation in that it is based on poll questions and is not a full-fledged study.

Several conclusions may be derived from these observations. As medical education continues to evolve, the student perspective will play an increasingly critical role in shaping how curricula are designed and implemented. The trends identified in these polls suggest that future MBBS programs may need to adapt to more student-centered approaches, where autonomy, regular feedback, and practical assessments take precedence.

Educators will need to strike a delicate balance between maintaining academic rigor and catering to the evolving preferences of students. By embracing self-directed learning, designing balanced assessments, and incorporating student feedback into teaching practices, medical schools can create an environment that not only prepares students for their professional exams but also fosters a lifelong commitment to learning.

In conclusion, the shift from passive to active learning models, coupled with a demand for more efficient assessment methods, reflects the changing mindset of medical students. As these trends continue to develop, they offer valuable insights into the future of medical education - one that is increasingly student-centered, technology-driven, and outcome-focused. Nevertheless, a need for more detailed studies on perception and challenges among medical students and faculties regarding small group learning is a need of the hour.
